# RELATIONSHIP CHANGES ACROSS THE CAREGIVING TRANSITION

**DOI:** 10.1093/geroni/igae098.1292

**Published:** 2024-12-31

**Authors:** Raquael Joiner, Niccole Nelson, Qimin Liu, Stacey Schepens Niemiec

**Affiliations:** University of Southern California, Los Angeles, California, United States; Colorado State University, Fort Collins, Colorado, United States; Boston University, Boston, Massachusetts, United States; University of Southern California, Los Angeles, California, United States

## Abstract

The majority of aging individuals’ care needs are met by family caregivers (CGs), often at great costs to CGs’ well-being. There is, however, significant heterogeneity in CG outcomes, and social capital, or the quality of marital, family, and friend relationships, is widely recognized as an im-portant factor underlying individual differences in CG outcomes. Because empirical work to date largely focuses on either cross-sectional differences or changes in social capital post-caregiving on-set, it is unclear, however, whether social capital disparities across different populations are a func-tion of the transition to CG or whether such disparities exist prior to taking on a CG role. Thus, we used data from the Health and Retirement Study (N=366; 8-waves) and Bayesian multi-phase latent growth curve models to examine social capital changes across the caregiving transition. Results sug-gested that CGs, on average, do not show significant changes in their social capital across the transi-tion to caregiving. Yet, there were significant individual differences, and individual differences in pre-caregiving social capital were significantly associated with social capital changes following tak-ing on a caregiving role: CGs with higher initial levels of social capital showed less declines in rela-tionship quality leading up to caregiving onset, but steeper declines in relationship quality at caregiv-ing onset and smaller relationship quality increases in the years following caregiving onset. This suggests that the social capital of individuals with initially satisfying relationships are most impacted by caregiving. We discuss the implications of these findings for refining and developing personal-ized social support interventions for CG populations.

